# The Effects of Variant Allele Frequency for EGFR Mutation on Early Tumor Shrinkage and Deepness of Response to Osimertinib in Patients with Metastatic Non-Small Cell Lung Cancer: An Exploratory Analysis

**DOI:** 10.3390/jcm15030944

**Published:** 2026-01-24

**Authors:** Giuseppe Bronte, Aldo Carnevale, Antonella Ciancetta, Donato Michele Cosi, Cristina Fragale, Stefania Ciarrocchi, Maria Luisa Di Guglielmo, Giovanna Tinelli, Noemi Mindicini, Lucia Battara, Lucilla D’Abundo, Elisa Callegari, Giovanni Lanza, Deborah Gabriele, Roberta Gafà, Alessandra Santini, Massimo Negrini, Luana Calabrò

**Affiliations:** 1 Department of Translational Medicine, University of Ferrara, 44121 Ferrara, Italycristina.fragale@edu.unife.it (C.F.); stefania.ciarrocchi@edu.unife.it (S.C.); elisa.callegari@unife.it (E.C.); giovanni.lanza@unife.it (G.L.); roberta.gafa@unife.it (R.G.);; 2 Unit of Oncology, University Hospital of Ferrara, 44124 Ferrara, Italy; 3 Department of Translational Medicine—Section of Radiology, University of Ferrara, 44121 Ferrara, Italy; aldo.carnevale@unife.it (A.C.);; 4 Department of Chemical, Pharmaceutical and Agricultural Sciences, University of Ferrara, 44121 Ferrara, Italy; 5 Laboratorio per le Tecnologie delle Terapie Avanzate, Tecnopolo, University of Ferrara, 44121 Ferrara, Italy; 6 Pathology Unit, Department of Oncology and Hematology, University Hospital of Ferrara, 44124 Ferrara, Italy

**Keywords:** epidermal growth factor receptor, variant allele frequency, non-small cell lung cancer, osimertinib

## Abstract

**Background:** Several studies evaluated the role of variant allele frequency (VAF) as a clinical decision-making tool for targeted therapies. However, its predictive role for treatment response in epidermal growth factor receptor (EGFR)-mutated non-small cell lung cancer (NSCLC) remains debated. This study investigates the relationship between VAF and early tumor shrinkage (ETS) and deepness of response (DpR). We also explored the impact of previously undescribed compound uncommon EGFR mutations on osimertinib activity. **Methods:** We retrospectively analyzed data from patients with advanced EGFR-mutated NSCLC, treated with osimertinib. VAF was obtained through NGS. We calculated corrected VAF (cVAF) based on the percentage of tumor cells. ETS and DpR were assessed according to RECIST 1.1 criteria. Molecular modeling was performed to predict the impact of novel compound EGFR mutations on osimertinib binding and EGFR protein structure. **Results:** We included 16 patients, who met the eligibility criteria. We found no significant correlation between cVAF and ETS or DpR, suggesting that cVAF may not have a direct effect on early or late tumor response to osimertinib. Median cVAF was 14%. Median progression-free survival and overall survival were longer in patients with higher VAF, even though they were not statistically significant. We identified two previously unreported compound EGFR mutations: N771Y + L858R and L718V + K713R + L858R. **Conclusions:** This study demonstrates that cVAF of EGFR mutations is not significantly associated with ETS or DpR during osimertinib in mNSCLC patients. Survival does not appear to be influenced by cVAF either. The identification and structural characterization of novel compound EGFR uncommon mutations may explain the benefit experienced by patients.

## 1. Introduction

Oncogene-addicted non-small cell lung cancer (NSCLC) represents around two-thirds of lung adenocarcinomas. Some of the altered oncogene drivers, i.e., EGFR, KRAS, MET, HER2, BRAF, ALK, ROS1, RET, and NTRK, can be targeted with a clear improvement in patient survival and safety, as compared to standard chemotherapy [1]. For non-oncogene-addicted NSCLCs, since there are no specific targets, the standard treatment is based on immune checkpoint inhibitors, alone or in combination with platinum-based chemotherapy, in agreement with programmed death ligand 1 (PD-L1) expression in tumor tissue [2].

The epidermal growth factor receptor (*EGFR*) gene usually bears mutations in the kinase domain (exons 18–21), leading to constitutive downstream EGFR signaling. Exon 19 deletion (ex19del) in amino acid residues 747 to 750 and exon 21 L858R point mutation are the most frequent (70–85%) EGFR activating mutations, so are defined as “common” or “classical” in newly diagnosed EGFR-mutant NSCLC. We can also find de novo T790M mutation, but rarely [3]. Other mutations in the kinase domain, which activate EGFR and are oncogenic, are defined as “uncommon” or “atypical”. In some patients, EGFR bears more than one mutation. We usually refer to this as “compound mutations”, which can include either two uncommon mutations or a common one with one or two uncommon mutations [4]. Many studies showed consistent efficacy of EGFR tyrosine kinase inhibitors (TKIs) in comparison with chemotherapy in advanced or metastatic EGFR-positive NSCLC. Osimertinib, a third-generation TKI, became the standard of care for the first-line treatment of these patients because of high tolerability and good central nervous system activity [5].

Tumor tissue remains the main source of tumor DNA for testing. Its detection via real-time PCR (qPCR) is being replaced by next-generation sequencing (NGS). This technique offers higher throughput, enabling the simultaneous sequencing of multiple genomic regions. As a result, a single run can detect various genetic alterations, including point mutations, insertions, deletions, and copy-number variations. Additionally, NGS is a quantitative method, providing read counts that allow for a sensitivity for detecting variant allele frequencies (VAF) as low as 3% [6].

Variant allele frequency (VAF) measures the fraction of alleles with a gene alteration: more specifically, it is a proportion of sequencing reads for a specific variant allele in relation to the total number of reads of a gene locus [7]. Thus, a high VAF percentage should associate with a high fraction of tumor cells harboring that gene alteration. Moreover, VAF may be useful to differentiate driver and passenger mutations. However, until now, it did not reach the reliable predictive value as an independent biomarker to select patients for target therapy [8]. For this reason, we explored whether VAF could be predictive of percentage tumor variation under EGFR-TKI treatment in EGFR-positive advanced NSCLC patients.

## 2. Materials and Methods

### 2.1. Study Design

We carried out this study as an exploratory retrospective analysis. We collected data from the clinical records of patients with a histologically confirmed diagnosis of unresectable locally advanced or metastatic (Stage IIIB–IV) NSCLC and treated with an EGFR-TKI in the last 5 years.

### 2.2. Study Objectives

The primary objective of the study aimed to explore the relationship between the corrected VAF (cVAF) of the main EGFR activating mutations with the early tumor shrinkage (ETS) and the deepness of response (DpR). VAF is the percentage measurement of allelic heterogeneity in a given genomic locus. When more than one mutation was present, for the analyses in this study, we considered the one with a higher value for VAF. Then, we calculated the cVAF, which considers the proportion of tumor cells (TC) in each specimen through the following formula: (VAF/TC) × 100. We calculated ETS as the percentage change in the sum of the maximum diameters of the target lesions according to the RECIST 1.1 criteria (Response Evaluation Criteria in Solid Tumors), at the time of first imaging evaluation after the start of treatment compared to the baseline evaluation. We calculated DpR as the percentage change in the sum of the maximum diameters of the target lesions according to the RECIST 1.1 criteria, at the time of the greatest reduction compared to the baseline evaluation.

The secondary objectives of the study were as follows:

(1) The association between the cVAF of the main activating EGFR mutation and best tumor response (BTR). We intended this to be the best response recorded from the start of the treatment until disease progression. For BTR, we used classification according to RECIST 1.1: complete response (CR), partial response (PR), stable disease (SD), and progressive disease (PD).

(2) The association between the cVAF of the main activating EGFR mutation and progression-free survival (PFS). We intended this to be the interval between the date of the start of EGFR-TKI until the date of increase in tumor mass of at least 20%, or appearance of new lesions, or death of the patient.

(3) The association between the cVAF of the main activating EGFR mutation and overall survival (OS). We intended this to be the time interval between the date of the start of EGFR-TKI until the date of death or last visit.

(4) Quality evaluation of molecular modeling to explain clinical outcomes, for uncommon mutations of unknown pathogenic meaning.

### 2.3. Eligibility Criteria

The patients were included for data collection and analyses if they met the following eligibility criteria:

- Histologically confirmed diagnosis of NSCLC;
- Non-squamous histology;
- AJCC (or TNM) Staging IIIB–IV;
- A finding by next generation sequencing (NGS) of at least one activating mutation of the *EGFR* gene, with report of VAF;
- At least one measurable lesion at serial contrast-enhanced CT scans, worth being taken into account as a target lesion for DpR measurement;
- A treatment with an EGFR-TKI for advanced disease;
- At least 6 months of follow-up since the start of this therapy.

### 2.4. Data Collection

We collected data from electronic clinical records. We extracted the following data for each patient: age at diagnosis, gender, Eastern Cooperative Oncology Group Performance Status (ECOG-PS), date of diagnosis, TNM staging and relative AJCC classification, sites of metastases, histology, previous surgery, previous radiotherapy, previous chemotherapy, type of EGFR-TKI, line of EGFR-TKI, date of start of EGFR-TKI, date of the end of EGFR-TKI, reason for the end of EGFR-TKI, date of disease progression, date of the last follow-up visit, date of death, date of biopsy, site of biopsy, type and VAF of each DNA mutation (common EGFR, uncommon EGFR, and other non-EGFR mutations), and the dates of the baseline and subsequent imaging assessments.

Radiologists with more than 10 years of experience retrieved CT scans from electronic imaging databases to determine ETS, DpR, and BTR retrospectively, as defined above.

### 2.5. Statistical Analyses

For the primary objective—that is, the association of continuous variables, i.e., cVAF, ETS, DpR—we calculated the correlation coefficient, which was associated with a graphical representation by scatter plot and regression line. For each continuous variable, we used the Shapiro–Wilk test for normal distribution. If normality was accepted, Pearson’s correlation coefficient would be used. For correlation analysis, a minimum sample size of 13 patients is required for a correlation coefficient of 0.7 with a statistical power of 80% and an alpha-error of 5%. A coefficient of 0.7 is usually interpreted as a good correlation [9].

For the association of cVAF and a categorical variable, i.e., BTR, first we calculated the median cVAF to make this a binomial variable. We chose the median as the cutoff point of cVAF to ensure equal distribution between the two groups. Subsequently, we tested this hypothesis by means of a chi-squared test.

For the association of cVAF and duration variables, i.e., PFS and OS, we used the median cVAF, which represents the cutoff for two subgroups (cVAF < or > median), which was useful for the analysis by the logrank test. To compare survival (PFS and OS) in these two subgroups, we calculated the hazard ratios (HR) and the related 95% confidence intervals (95% CI) via the Cox proportional hazards regression.

For all statistical tests, we considered *p* < 0.05 to be statistically significant. We performed statistical analyses by means of MedCalc version 22.023 (MedCalc Software Ltd., Ostend, Belgium).

### 2.6. Molecular Modeling Analysis

Three-dimensional models of wild-type and N771Y/L858R and L718V/K713R/L858R mutant EGFR tyrosine kinase domain (P694-G1021 portion) were built using an AlphaFold 3 web-server [10] by selecting ADP as co-folding ligand. Osimertinib coordinates were imported in the models by superimposing them onto the osimertinib X-ray complex with the mutant L858R/T790M/C797S EGFR available in the Protein Data Bank (PDB ID: 6LUD) [11] by preserving key drug–target interactions observed in the experimental structure and ensuring no significant clashes were introduced. Osimertinib–EGFR interaction analysis was carried out with PLIP [12] and the images were rendered with PyMOL open source v2.5.0 (Schrödinger, Inc., New York, NY, USA).

## 3. Results

### 3.1. Baseline Patients’ Characteristics

We included 16 patients from our center (Oncology Unit of the University Hospital of Ferrara, Italy) who met all the eligibility criteria. The mean age was 67 (range 49–80). The female-to-male ratio was 1:1. Eighty-one percent of patients had ECOG PS 0-1. Eighty-eight percent had AJCC stage IVB, and those remaining had stage IVA. All patients had adenocarcinoma as a histotype. The sites of metastases included the following: bone (13 patients), lung (12), pleura (4), CNS (3), liver (2), distant lymph nodes (2), and adrenal gland (1). Two patients received previous radiotherapy as a palliative treatment for symptomatic metastases and one patient received chemotherapy before EGFR-TKI, because the mutation test was not immediately available and the neoplastic disease was symptomatic. All patients received osimertinib as EGFR-TKI (Supplementary Table S1). Only two patients were forced to stop the therapy because of toxicity. The time of first imaging evaluation for the calculation of ETS was different among the patients with a mean time of 4.9 months.

### 3.2. Mutation Profiling

In all patients, tumor tissue from biopsy was assessed through NGS gene profiling to find targetable driver mutations. All NGS analyses were performed at our center. Sequencing analysis was conducted on DNA extracted from formalin-fixed, paraffin-embedded (FFPE) tissue samples. Library preparation and sequencing were performed using the Oncomine™ Precision Assay on the Genexus™ Integrated Sequencer (Ion Torrent platform), in accordance with the manufacturer’s protocols. Bioinformatic analysis of sequencing data was carried out using the integrated Genexus™ Dx Software version 6.6.

**Figure 1:**
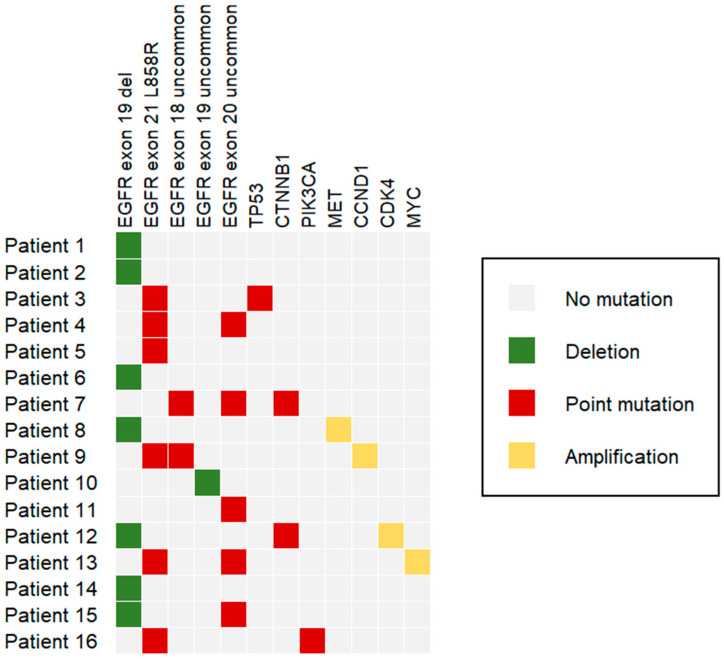
Genomic profiling of the patients included in this study (heatmap). We used different colors to distinguish the type of gene alteration (deletion, point mutation, amplification).

The VAF percentage was reported in the record, together with the type of mutation. Among the 16 patients included, 7 had EGFR exon 19 deletion and 6 had EGFR exon 21 L858R. The other patients had uncommon mutations (Figure 1, Supplementary Table S2). In patients with compound mutations, the VAF percentage for each mutation was similar; this suggested an in-cis occurrence (Supplementary Table S2).

### 3.3. Association Between cVAF and ETS or DpR

We collected all the measures of target lesions at the baseline and all subsequent reassessment CT scans. We graphed these data as percentage variation in the sum of the maximum diameters of the target lesions from the baseline (Supplementary Figure S1). In all patients, except for Patient 7, a reduction in the tumor volume occurred at the time of imaging analyses following the start of therapy. Patient 7, however, continued the treatment for clinical benefit in terms of symptom improvement, despite a constant increase in the sum of the maximum diameters of the target lesions, without new lesions.

**Figure 2:**
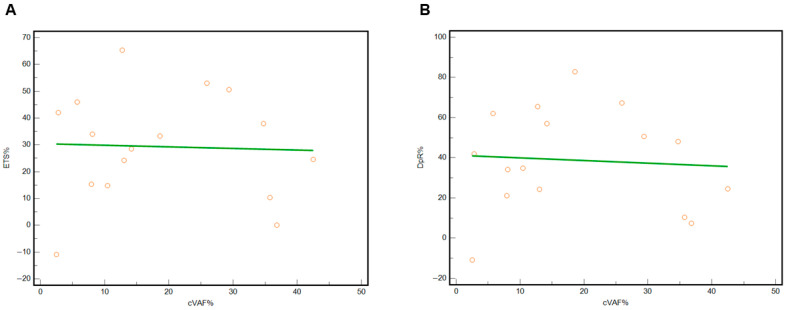
(**A**) Correlation between cVAF (independent variable, x axis) and ETS (dependent variable, y axis) and (**B**) correlation between cVAF (independent variable, x axis) and DpR (dependent variable, y axis). The green line is a linear regression fit; circles represent the relationship between cVAF and ETS or DpR for each patient.

The distribution of the three variables, i.e., cVAF, ETS, and DpR, was normal. For this reason, we performed Pearson’s correlation analysis. We found no correlation between cVAF (independent variable) and ETS (dependent variable) for the value of the correlation coefficient, r = −0.04, *p* = 0.89 (Figure 2A). Similarly, we found no correlation between cVAF (independent variable) and DpR (dependent variable), for the value of the correlation coefficient, r = −0.07, *p* = 0.80 (Figure 2B). We graphed these findings through the scatter diagram with a regression line.

### 3.4. Association Between cVAF and Other Outcome Measures

We estimated BTR according to RECIST 1.1. We found no CR; seven patients who experienced PR as BTR; four patients who experienced SD; and in those who remained, we observed PD. We calculated the median cVAF and we used this value as a cutoff. The median value of cVAF was 14%. Consequently, we analyzed the association between BTR in these three categories (PR, SD, PD) and the two groups, according to VAF < or > its median. The majority of patients experiencing PD as BTR (80%) had cVAF higher than the median. The majority of patients experiencing PR (57%) and SD (75%) had cVAF lower than the median (Supplementary Figure S2). However, this inverse relationship was not statistically significant. The chi-squared value was 2.94, *p* = 0.23.

**Figure 3:**
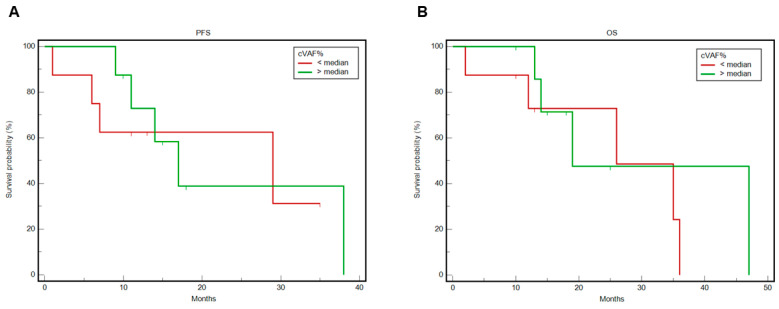
Kaplan–Meier curves for the comparison of PFS (**A**) and OS (**B**) in the two subgroups, according to median cVAF.

In the overall population, the median PFS was 17 months (95% CI: 9–38), whereas the median OS was 26 months (95% CI: 13–36). The 12-month OS rate was 86.5% (95% CI: 55.8–96.5%). Specifically, it was 100% (95% CI: 0.6–91.0%) in the subgroup with stage IVA, and 84.4% (95% CI: 50.4–95.9%) in those with stage IVB. A cVAF percentage that was equal to or greater than the median was associated with shorter median PFS compared to a lower percentage: 17 months (95% CI: 9–38) vs. 29 months (95% CI: 1–29); HR: 1.18 (95% CI: 0.29–4.86) and *p* = 0.82 (Figure 3A). This wide difference in median survival suggests that a prospective study with more patients may help us to reach statistical significance. Accordingly, a cVAF percentage that was equal to or greater than the median was associated with shorter median OS: 19 months (95% CI: 13–47) vs. 26 months (95% CI: 2–36); HR: 1.53 (95% CI: 0.37–6.37) and *p* = 0.56 (Figure 3B).

### 3.5. Molecular Modeling

**Figure 4:**
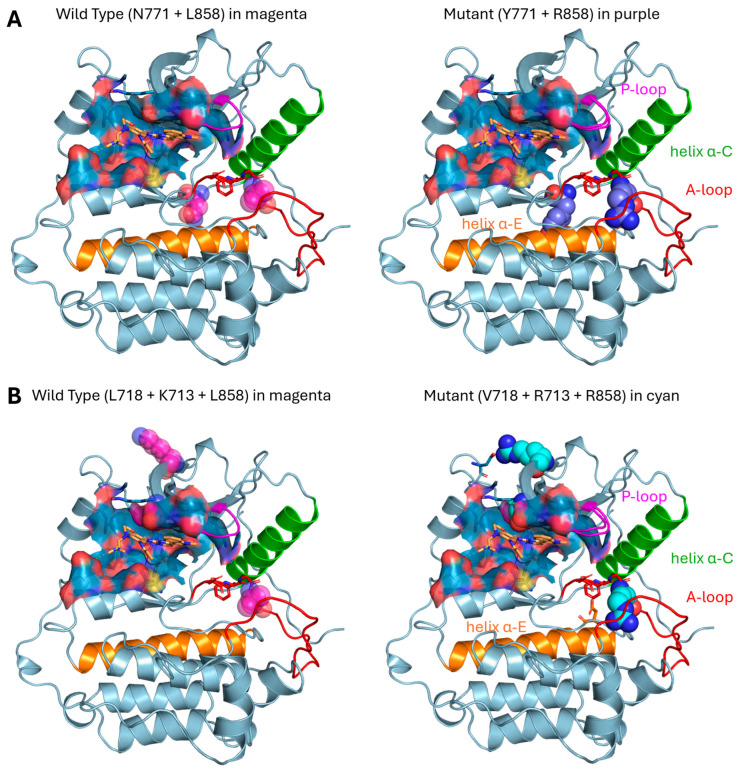
Molecular modeling of EGFR protein with compound mutations bound to osimertinib. (**A**) Both N771Y and L858R mutations (purple spheres) are located far from the active site (blue surface) and do not interfere with drug binding (orange sticks). These mutations are located near the loop that follows the α-C helix (green) and within the A-loop (red), creating a steric hindrance that presumably pushes and stabilizes the α-C helix in the “helix-out” conformation and interferes with the A-loop function. (**B**) EGFR protein with triple mutations L718V, K713R, and L858R. Among these three mutations, only V718 is located near the active site (blue surface), but this does not seem to have a measurable effect on drug binding (orange sticks) because it is slightly bulkier than Leucine. R713 is located in a position that we do not think interferes with either drug or enzymatic activity, although it is located at a distance that is compatible with an electrostatic interaction with Asp1006, which could subtly modulate the P-loops dynamics to favor an orientation that is capable of hosting osimertinib. R858, on the contrary, as already known, creates steric hindrance within the A-loop (red), which presumably enforces a different loop conformation. Therefore, this interferes with the enzymatic function without preventing the drug from binding and performing its function. Note the possibility of the formation of a salt-bridge with either Asp 837 (orange sticks near R858) or Asp855 (red sticks near R858), which can stabilize the enzyme in a conformation that has not yet experimentally been observed but is thoroughly characterized by high level molecular dynamics [18] and conformational free energy landscape [19] studies, suggesting an electrostatic rewiring where R858 engages in alternative salt-bridges with acidic residues of the activation loop and lowers the barriers for non-canonical orientations that have not yet been observed in static structures.

Among the mutations described in the Supplementary Table S1, some were compound mutations including multiple uncommon mutations, or a common one with one uncommon mutation, which have never been described in the literature. Thus, the relative pathogenic significance and effects on sensitivity to the drug osimertinib are unknown. Therefore, for Patient 4 (N771Y + L858R) and for Patient 9 (L718V + K713R + L858R), molecular modeling of the EGFR mutant protein was performed. The position of the (N771Y + L858R) and (L718V + K713R + L858R) mutations (purple and cyan spheres in Figure 4A,B right panels, respectively) with respect to the osimertinib binding site (blue surface in Figure 4, ligand in orange sticks) and their putative effect, with respect to the wild type structure (WT residues highlighted with magenta spheres in Figure 4A,B left panels) in the overall kinase domain architecture were inspected.

As detailed in Figure 4A (right panel), both N771Y and L858R mutations were located distal to the drug binding pocket (blue surface, osimertinib in orange sticks): the N771Y mutation insisted on the loop following the α-C helix (green cartoons in Figure 4), whereas the L858R mutation was located on the activation loop (A-loop, red cartoons in Figure 4) right after the DFG motif (red sticks in Figure 4). Both locations were expected to negatively impact EGFR activation whilst still enabling osimertinib to be accommodated in the active site and exert its function. In particular, the Y771 residue was predicted to create steric bulk that might push the α-C helix away from its position and stabilize it in a “helix-out” conformation. The R858 residue, on the other hand, was expected to alter the electrostatic environment in the proximity of the DFG motif, by disrupting hydrophobic contacts within the A-loop and creating salt-bridges with negatively charged residues, such as D855 (orange sticks in Figure 4B, right panel) and D837 (red sticks in Figure 4), which were both located in the residue proximity. Notably, in a previous MD study, salt-bridges involving R858 were detected [13] and a further MD study highlighted the possibility that the L858R mutation might favor EGFR conformations that have not been experimentally observed yet [14]. Patient 4, who harbored this compound mutation, experienced PR as BTR.

As depicted in Figure 4B (right panel), among the co-occurring three mutations, only the L718V mutant was located in the proximity of the osimertinib binding site (blue surface in Figure 4, osimertinib in orange sticks) and positioned upstream the P-loop (magenta cartoons in Figure 4). L718 mutations (such as L718V and L718Q) are established mechanisms of acquired resistance to osimertinib [15,16]. Although valine has a smaller molecular volume than leucine, its β-branching and reduced hydrophobic contacts were proposed to stabilize a non-reactive conformation of the osimertinib acrylamide moiety whilst not preventing drug binding, as suggested by QM/MM studies [17]. In our model (Figure 4B, right panel), the P-loop was predicted to be pushed upwards, with respect to the wild type enzyme (Figure 4B, left panel), to accommodate the inhibitor. We speculated that this loop orientation was stabilized by the K713R mutation, where R713 lay at a distance (4.40 Å) that was compatible with an electrostatic interaction with a nearby acidic residue (D1006). The primary L858R mutation was expected to further impact the global enzyme dynamics, as previously discussed. Overall, we hypothesized that these complex mutations induced structural changes on the enzyme that shifted the equilibrium towards a α-C helix-out conformation and interfered with the A-loop position and function. Crucially, these changes appeared to impact the enzyme activity whilst still allowing for osimertinib to be accommodated in the active site and exert its function via P-loop repositioning. We proposed that the clinical outcome of Patient 9, who harbored this compound mutation, had PR as BTR, and experienced a prolonged duration of treatment with osimertinib (around 3 years), may be explained by the allosteric effect of the K713R mutation. By modulating the P-loop orientation, this mutant might prevent the full steric exclusion of osimertinib and maintain the enzyme in a drug-accessible state by partially mitigating the resistance typically conferred by the L718V mutation.

## 4. Discussion

EGFR-TKIs achieved a high efficacy in NSCLC patients, with EGFR activating mutations. EGFR exon 20 insertions apart, osimertinib reached the position of gold standard as upfront therapy in these patients [5]. Recently, combination strategies with other drugs emerged as possibly more effective treatment options, as shown in the FLAURA-2 and MARIPOSA trials [20,21].

Among the extensive data obtained through NGS, VAF is poorly used in clinical practice, even though many researchers have explored its possible prognostic or predictive role. First, VAF of the acquired T790M mutation in tumor tissue was explored to predict the efficacy of osimertinib as a subsequent treatment after first- or second-generation EGFR-TKIs. The authors found that the type of first-line EGFR-TKI matters, and higher allele frequency of T790M mutation is associated with longer PFS [22]. Conversely, a high VAF of EGFR activating and T790M mutations in circulating tumor DNA (ctDNA) were associated with shorter PFS and OS in NSCLC patients treated with EGFR-TKIs [23,24]. Similarly, some authors found a prognostic role for VAF, related to both *EGFR* and *KRAS* mutation in ctDNA from NSCLC patients [25]. Subsequently, some authors evaluated T790M allele frequency relative to EGFR activating mutations in matched plasma and tissue samples. They observed that patients with a low relative T790M allele frequency harbored more concomitant resistance gene alterations, e.g., *MET* amplification and *ERBB2* amplification, and experienced a poorer response to osimertinib [26]. Giezser et al. showed that patients harboring the EGFR exon 19 mutation in tumor tissue treated with gefitinib or erlotinib achieved significantly improved PFS and OS compared to those with exon 21 mutation. Moreover, VAF of the former EGFR mutation higher than 70% independently predicted longer PFS and OS [27]. Friedlaender et al. obtained similar results in a similar study [28].

None of these studies evaluated all patients treated with osimertinib as ours does. Moreover, we tried to verify whether cVAF is related to the change in tumor size in terms of ETS and DpR. First, some studies on advanced colorectal cancer evaluated these parameters. The authors defined the former as “the relative change in the sum of longest diameters of RECIST target lesions at week 8 compared with baseline”, and the latter as “the relative change in the sum of the longest diameters of RECIST target lesions at the nadir, compared with baseline” [29,30]. In NSCLC, a pooled analysis found that a greater DpR is associated with longer PFS and OS in treatments including ALK inhibitors or anti-PD-1 drugs [31]. However, no studies investigated the correlation of these size variables with cVAF in NSCLC patients. Our findings help us to think that we can achieve tumor shrinkage regardless of a high cVAF, even though a greater patient sample size would have reached a stronger statistical power for this conclusion.

Among the EGFR mutations detected through NGS, we found two patients harboring compound mutations, which no authors have described before. These mutations are composed of a common mutation (exon 21 L858R) with other uncommon mutations. We compared our cases with a systematic review (1836 patients from 38 studies) and a translational study (16,715 patients from five databases) [3,4]. For this reason, we modeled these two cases of compound mutations in the EGFR protein to speculate about the biological meaning through the structural changes. According to the structure-based classification proposed by Robichaux et al. [3], these two compound mutations cannot be included in any of the four structure-based categories. However, in both cases, the structural changes do not hamper osimertinib from being accommodated in the active site and exerting its function. This speculation can also explain the outcome of osimertinib in both of the patients harboring these mutations.

This study also has some limitations. The sample size is insufficient to draw conclusions on the correlation between cVAF and ETS or DpR, which was the primary objective of the study. For this reason, the study likely has low statistical power to detect a biological association. Moreover, the lack of statistical significance does not prove that no relationship exists, but the results may suggest this. This study included patients with real-world experience. They mainly had common mutations and some of them had uncommon ones. The differences between common and uncommon mutations was not an aim for this study. Combining these mutation groups may mask specific effects within subgroups. For these reasons, the nature of this retrospective study remains exploratory, without the possibility of bringing definitive results.

In conclusion, this exploratory work highlighted that cVAF of EGFR mutations may not influence the change in tumor size, in terms of ETS and DpR. Other studies found an association of VAF with survival outcomes, such as PFS and OS. These apparently conflicting findings may mean that osimertinib mainly achieves its effect regardless of the amount of mutated cells. However, many factors can influence this result. We performed the molecular modeling of compound mutations, not described before, to show that osimertinib can act on its target even in these cases. Since this is an exploratory retrospective study with a small patient population, a larger prospective trial can be designed to corroborate these findings.

## Data Availability

The datasets generated and/or analyzed during the current study are available from the corresponding author upon reasonable request.

## Supplementary Materials

The following supporting information can be downloaded at: https://www.mdpi.com/article/10.3390/jcm15030944/s1, Supplementary Figure S1: Percentage changes in the sum of the maximum diameters of the target lesions from baseline for each patient; Supplementary Figure S2: Distribution of BTR categories (PR, SD, PD) in the two subgroups according to median VAF; Supplementary Table S1: Baseline characteristics of the patients; Supplementary Table S2: Type of mutations and variant allele frequency for each patient.

## Abbreviations

The following abbreviations are used in this manuscript:

BTR	Best Tumor Response
CI	Confidence Intervals
CNS	Central Nervous System
CR	Complete Response
CT	Computed Tomography
ctDNA	Circulating Tumor DNA
cVAF	Corrected Variant Allele Frequency
DpR	Deepness of Response
ECOG-PS	Eastern Cooperative Oncology Group Performance Status
EGFR	Epidermal Growth Factor Receptor
ETS	Early Tumor Shrinkage
HR	Hazard Ratio
mNSCLC	Metastatic Non-Small Cell Lung Cancer
NGS	Next-Generation Sequencing
NSCLC	Non-Small Cell Lung Cancer
OS	Overall Survival
PCR	Polymerase Chain Reaction
PD	Progressive Disease
PD-L1	Programmed Death Ligand 1
PFS	Progression-Free Survival
PR	Partial Response
qPCR	Quantitative Polymerase Chain Reaction
RECIST	Response Evaluation Criteria in Solid Tumors
SD	Stable Disease
TC	Tumor Cells
TKI	Tyrosine Kinase Inhibitor
VAF	Variant Allele Frequency
WT	Wild-Type
