# Supplementary material for: The Effects of Variant Allele Frequency for EGFR Mutation on Early Tumor Shrinkage and Deepness of Response to Osimertinib in Patients with Metastatic Non-Small Cell Lung Cancer: An Exploratory Analysis
Source: J Clin Med. 2026 Jan 24;15(3):944. doi: 10.3390/jcm15030944 (PMC12898104; doi:10.3390/jcm15030944)
Supplement: Supplementary file 1 [file jcm-15-00944-s001.zip › jcm-4076769-supplementary.pdf]

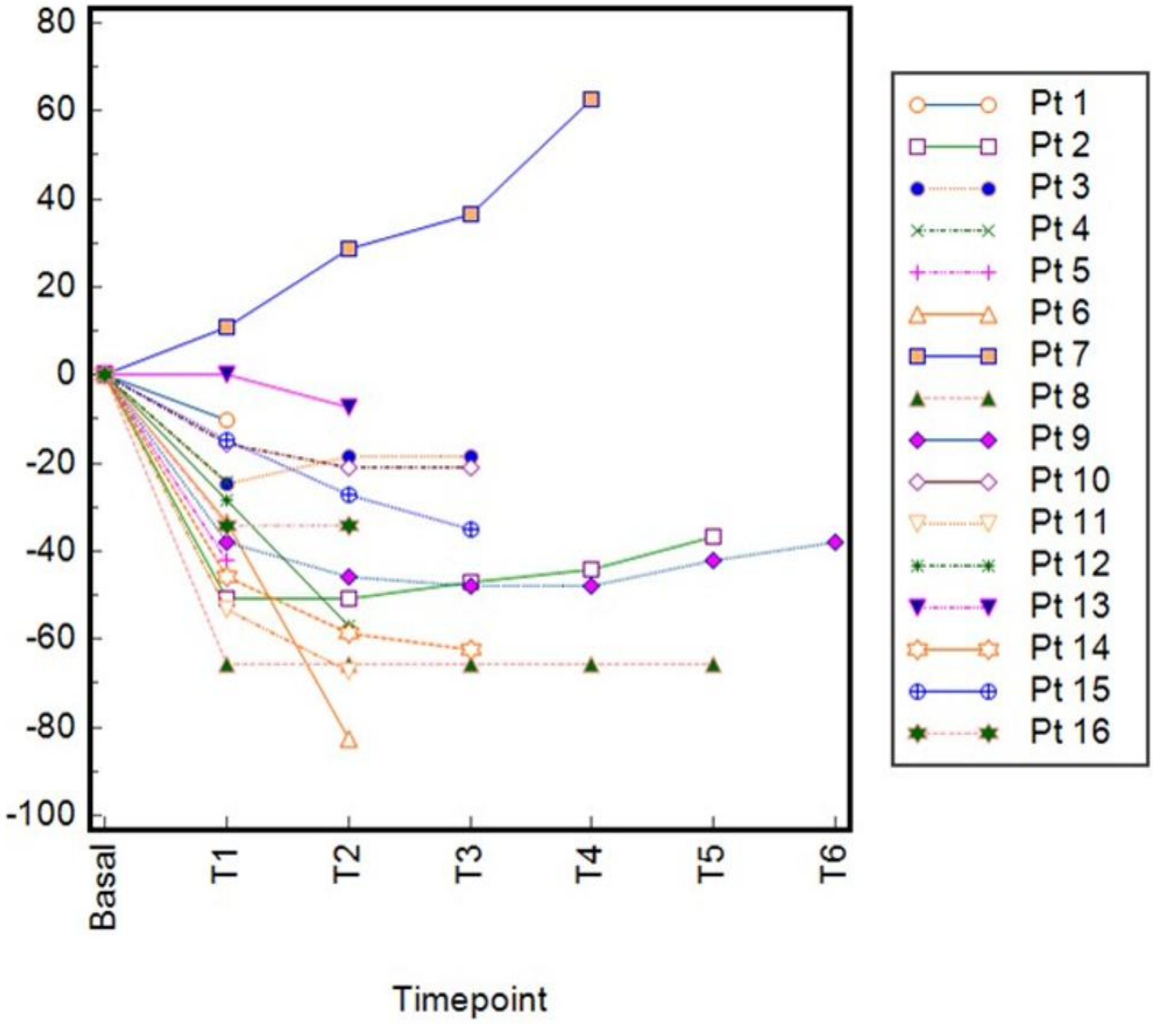

Supplementary Figure S1.

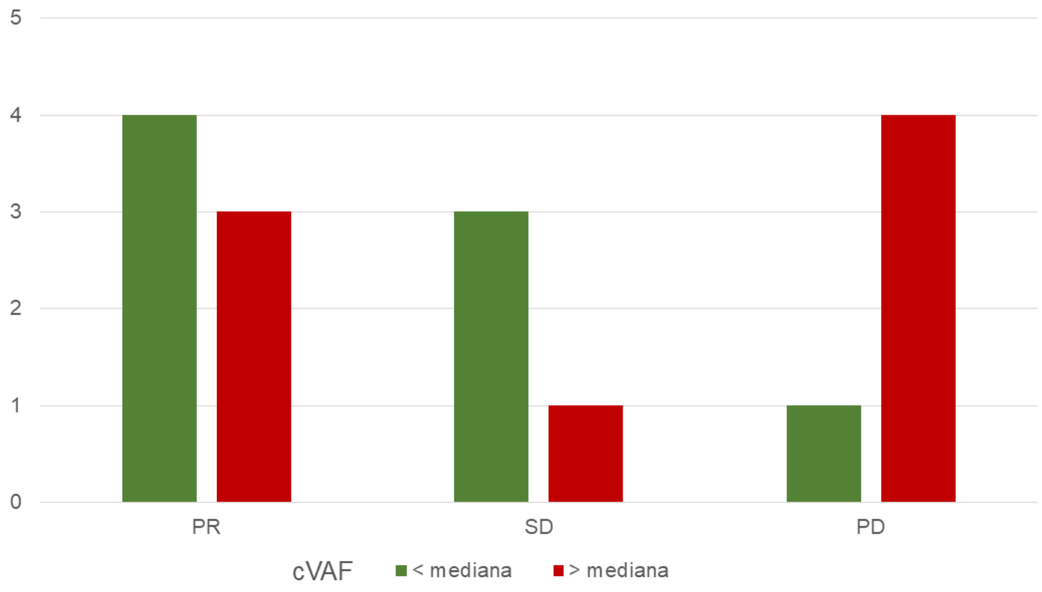

Supplementary Figure S2.

Characteristic	Category	Value
Age	Mean	67
	Range	49 - 80
Gender	Female	8
	Male	8
ECOG Performance Status	0	3
	1	10
	2	2
	3	1
	4	0
AJCC Stage	IVA	2
	IVB	14
Histotype	Adenocarcinoma	16
	Other	0
Previous Radiotherapy	Yes	2
	No	14
Previous Chemotherapy	Yes	1
	No	15
EGFR TKI	Osimertinib	16
	Other	0

Supplementary Table S1.

Patient #	EGFR Common mutations (VAF%)	EGFR Uncommon mutations (VAF%)	Other genes (VAF%)
Patient 1	Exon 19 deletion (71%)		
Patient 2	Exon 19 deletion (49%)		
Patient 3	Exon 21 L858R (47%)		TP53 - Q192* (74%)
Patient 4	Exon 21 L858R (22%)	Exon 20 N771Y (28%)	
Patient 5	Exon 21 L858R (7%)		
Patient 6	Exon 19 deletion (31%)		
Patient 7		Exon 18 G719S (8%)<br>Exon 20 S768I (8%)	CTNNB1 Exon 3 S33F (3%)
Patient 8	Exon 19 deletion (43%)		MET amplification 7q31.2
Patient 9	Exon 21 L858R (58%)	Exon 18 L718V (40%)<br>Exon 18 K713R (40%)	CCND1 amplification 11q13.3
Patient 10		Exon 19 L747-P753delinsS (27%)	
Patient 11		Exon 20 S768I (43%)	
Patient 12	Exon 19 deletion (35%)		CTNNB1 Exon 3 G34E (4%)<br>CDK4 amplification 12q14.1
Patient 13	Exon 21 L858R (46%)	Exon 20 T790M (47%)	MYC amplification 2p24.3 6.3x
Patient 14	Exon 19 deletion (19%)		
Patient 15	Exon 19 deletion (26%)	Exon 20 T790M (27%)	
Patient 16	Exon 21 L858R (20%)		PIK3CA Exon 8 C420R (10%)

Supplementary Table S2.
